# A basic analysis toolkit for biological sequences

**DOI:** 10.1186/1748-7188-2-10

**Published:** 2007-09-18

**Authors:** Raffaele Giancarlo, Alessandro Siragusa, Enrico Siragusa, Filippo Utro

**Affiliations:** 1 Dipartimento di Matematica Applicazioni, Università di Palermo, Italy

## Abstract

This paper presents a software library, nicknamed BATS, for some basic sequence analysis tasks. Namely, local alignments, via approximate string matching, and global alignments, via longest common subsequence and alignments with affine and concave gap cost functions. Moreover, it also supports filtering operations to select strings from a set and establish their statistical significance, via z-score computation. None of the algorithms is new, but although they are generally regarded as fundamental for sequence analysis, they have not been implemented in a single and consistent software package, as we do here. Therefore, our main contribution is to fill this gap between algorithmic theory and practice by providing an extensible and easy to use software library that includes algorithms for the mentioned string matching and alignment problems. The library consists of C/C++ library functions as well as Perl library functions. It can be interfaced with Bioperl and can also be used as a stand-alone system with a GUI. The software is available at  under the GNU GPL.

## 1 Introduction

Computational analysis of biological sequences has became an extremely rich field of modern science and a highly interdisciplinary area, where statistical and algorithmic methods play a key role [1,2]. In particular, sequence alignment tools have been at the hearth of this field for nearly 50 years and it is commonly accepted that the initial investigation of the mathematical notion of alignment and distance is one of the major contributions of S. Ulam to sequence analysis in molecular biology [3]. Moreover, alignment techniques have a wealth of applications in other domains, as pointed out for the first time in [4].

**Figure 1:**
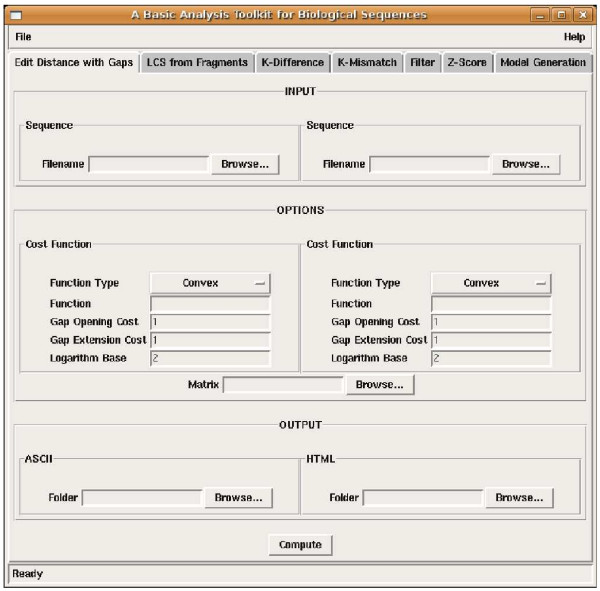
**a snapshot of the GUI**. An overview of the GUI of BATS. The top bar has a specific button for each of the algorithms and functions implemented. Then, each function has its own parameter selection interface. The Edit Distance function interface is shown here.

Here we concentrate on alignment problems involving only two sequences. In general, they can be divided in two areas: local and global alignments [1]. Local alignment methods try to find regions of high similarity between two strings, e.g. BLAST [5], as opposed to global alignment methods that assess an overall structural similarity between the two strings, e.g. the Gotoh alignment algorithm [6]. However, at the algorithmic level, both classes often share the same ideas and techniques, being in most cases all based on dynamic programming algorithms and related speed-ups [7]. More in detail, we have implementations for (see also Fig. 1 for the corresponding function in the GUI):

(a) Approximate string matching with *k *mismatches. That is, given a pattern and text string and an integer *k*, we are interested in finding all occurrences of the pattern in the text with at most *k *mismatching characters per occurrence. We provide an implementation of an algorithm given in [8]. It is a simplification of the first efficient algorithm obtained for this problem, due to Landau and Vishkin [9]. The asymptotically fastest known algorithm to date is due to Amir, Lewenstein and Porat [10]. Formalization of the problem, as well as description of the algorithm and library functions, both in C/C++ and Perl, is given in section 2.

(b) Approximate string matching with *k *differences. That is, given a pattern and text string and an integer *k*, we are interested in finding all occurrences of the pattern in the text with at most *k *differences where, for each occurrence a "difference" is a character to be inserted, deleted or substituted in the pattern. We provide an implementation of the algorithm by Landau and Vishkin [11], although the asymptotically most efficient one, to date, has been recently obtained by Cole and Hariharan [12]. Formalization of the problem, as well as description of the algorithm and library functions, both in C/C++ and Perl, is given in section 3.

(c) The longest common subsequence from fragments, a generalization of the well known longest common subsequence problem [1], considered by Baker and Giancarlo [13]. Formalization of the problem, as well as description of the algorithm and library functions, both in C/C++ and Perl, is given in section 4.

(d) Edit distance with concave and affine gap penalties. It is the well known generalization of the edit distance between two strings introduced by M.S. Waterman [14], i.e., with the use of concave gap costs. We provide an implementation of the algorithm obtained by Galil and Giancarlo [15] (**GG **algorithm for short). An analogous algorithm was obtained independently by Miller and Myers [16]. We also point out that the asymptotically most efficient algorithm, to date, is still the one given by Klawe and Kleitman [17], although it seems to be mainly of theoretic interest. It is also worth mentioning that the **GG **algorithm naturally specializes to deal with affine gap costs. Formalization of the problem, as well as description of the algorithm and library functions, both in C/C++ and Perl, is given in section 5.

(e) Filtering, statistical significance computation and organism model generation. The first two functions allow to select a subset of strings from a given set and to assess its statistical significance via z-score computation [18]. The third function is required in order to give to the first two, a probabilistic model of the input data. While the filtering techniques are quite standard, the implementation of the z-score computation is a specialization of a non-trivial implementation by Sinha and Tompa, used for motif discovery [19]. Our code, as the one by Sinha and Tompa, works only for DNA sequences. The function allowing for the generation of a user-specified model organism gives, in a suitable format, all probabilistic information needed by the z-score function. Description of this part of the system, as well as presentation of the corresponding library functions, both in C/C++ and Perl, is given in section 6.

As it is self-evident from the description just given, this software library is not intended as a generic programming environment, like Leda for combinatorial and geometric computing [20]. An initial attempt, in that direction, for string algorithms is described in [21]. The software presented here is more tailored at specific alignment problems. We also point out that most of the algorithms implemented in BATS are based on suffix trees [22]. Here we use the algorithm by Ukkonen [23] in the Strmat library [24]. It is not particularly memory-efficient (17 bytes/character) and that may be problematic for genome-wide applications of the corresponding algorithms. We finally point out that the entire library can be used as a stand-alone system with a GUI and it can be interfaced with Bioperl. A detailed user manual, together with installation procedures, file formats etc., is given at the supplementary web site [25].

## 2 Approximate string matching with *k *mismatches

Given a text string *text *= *t*[1, *n*], a pattern string *pattern *= *p*[1, *m*] and an integer *k*, *k *≤ *m *≤ *n*, we are interested in finding all occurrences of the pattern in the text with at most *k *mismatches, i.e. with at most *k *locations in which the pattern and a text substring have different symbols.

Let *Prefix*(*i*, *j*) be a function that returns the length of the longest common prefix between *p*[*i*, *m*] and *t*[*j*, *n*]. It can be computed in *O*(1) time, after the following preprocessing step: (A) build the suffix tree *T *[22] of the strings *p*[1, *m*]$*t*[1, *n*], where $ is a delimiter not appearing anywhere else in the two strings; (B) preprocess *T *so that Lowest Common Ancestor (LCA for short) queries can be answered in constant time [26]. The preprocessing step takes *O*(*n *+ *m*) time and it is well known that the computation of *Prefix*(*i*, *j*) reduces to the computation of one LCA query on the leaves of *T *[8].

Once that the preprocessing step is completed, we can find the first (leftmost) mismatch between *p*[1, *m*] and *t*[*j*, *j *+ *m *- 1] in *O*(1) time by use of *Prefix*(1, *j*). If we keep track of where this mismatch occurs, say

1: Algorithm **SM**

2: **for ***j *= 1 **to ***n ***do**

3:    *pt *← *j*; *v *← 1; *num_mismatch *← 0;

4:    **t[*j*, *j *+ *m *- 1] is aligned with *p*[1, *m*] and no mismatch has been found**

5:    **while ***v *≤ *m *- 1 **and ***num_mismatch *≤ *k ***do**

6:

7:       **find leftmost mismatch between *t*[*pt*, *pt *+ *m *- 1] and *p*[*v*, *m*]**

8:       ℓ ← *Prefix*(*v*, *pt*)

9:       **if ***v *+ ℓ ≤ *m ***then**

10:          *num_mismatch *← *num_mismatch *+ 1

11:       **end if**

12:       *pt *← *pt *+ ℓ + 1; *v *← *v *+ ℓ + 1;

13:    **end while**

14:    **if ***num_mismatch *≤ *k ***then**

15:       **found match**

16:    **end if**

17: **end for**

at position *l *of *pattern*, we can locate the second mismatch, in *O*(1) time, by finding the leftmost mismatch between *p*[*l *+ 1, *m*] and *t*[*j *+ *l *- 1, *j *+ *m *- 1]. In general, the *q*-th mismatch between *p*[1, *m*] and *t*[*j*, *j *+ *m *- 1] can be found in *O*(1) time by knowing the location of the (*q *- 1)-th mismatch. Algorithm **SM **gives the needed pseudo-code. We have:

**Theorem 2.1 **[8,9]*Given a pattern p and a text t of length m and n respectively, Algorithm ***SM ***finds all occurrences of p in t with at most k mismatches in O*(*m *+ *n *+ *nk*) *time, including the preprocessing step*.

### 2.1 The C/C++ library functions

The function below returns all occurrences, with at most *k *mismatches, of a pattern within a text.

Synopsis

#include "k_mismatch.h"

OCCURRENCES

**k_mismatch**(char*text, char*pattern, int *k*);

**Arguments**:

• *text*: points to a text string;

• *pattern*: points to a pattern string;

• *k*: is an integer giving the maximum number of allowed mismatches.

**Return Values**: **k_mismatch** returns a pointer to OCCURRENCES_STRUCT, defined as:

typedef struct occurrences

{

   int* start*, *end*;

   int* errors*;

   char*text;

   char*pattern;

struct occurrences*next;

} OCCURRENCES_STRUCT, *OCCURRENCES;

where:

• *start*: is the start position of this occurrence in the text string;

• *end*: is the end position of this occurrence in the text string;

• *errors*: the number of mismatches of this occurrence;

• *text*: is a pointer to the aligned substring corresponding to the occurrence found;

• *pattern*: is a pointer to the aligned pattern string.

### 2.2 The PERL library functions

The function below returns all occurrences, with at most *k *mismatches, of a pattern within a text.

Synopsis

use BSAT::K_Mismatch;

K_Mismatch *Text Pattern K*

**Arguments**:

• *Text*: is a scalar containing the text string;

• *Pattern*: is a scalar containing the pattern string;

• *K*: is a scalar giving the maximum number of allowed mismatches.

**Return values: **The function returns an array of occurrences. Each occurrence consists of a hash:

my %occurrence = (

   errors => 0,

   start => 0,

   end => 0,

   text => "",

   pattern => "");

where the above fields are as in the OCCURRENCES_STRUCT defined earlier.

## 3 Approximate string matching with *k *differences

In this section we consider a more general problem of approximate string matching by extending the set of allowed differences between strings. Letting *text*, *pattern *and *k *be as in section 2, we are interested in finding all occurrences of *pattern *in *text *with at most *k *differences. The allowed differences are:

(a) A symbol of the pattern corresponds to a different symbol of the text, i.e., a mismatch.

(b) A symbol of the pattern corresponds to no symbol in the text.

(c) A symbol of the text corresponds to no symbol in the pattern.

Let *A *be an (*m *+ 1) × (*n *+ 1) dynamic programming matrix and consider the following recurrence:

*A*[0, *j*] = 0, 0 ≤ *j *<*n*.

*A*[*i*, 0] = *i*, 0 ≤ *i *<*m*.

*A*[*i*, *j*] = *min*(*A*[*i *- 1, *j*] + 1, *A*[*i*, *j *- 1] + 1, *if p*[*i*] = *t*[*j*] *then A*[*i *- 1, *j *- 1] *else A*[*i *- 1, *j *- 1] + 1).

Matrix *A *can be computed row by row, or column by column, in *O*(*nm*) time. Moreover, it can be easily shown that *A*[*i*, *j*] is the minimal edit distance between *p*[1, *i*] and a substring of *text *ending at position *j*. Thus, it follows that there is an occurrence of the pattern in the text ending at position *j *of the text if and only if *A*[*m*, *j*] ≤ *k*. The computation of *A *can be substantially sped-up by observing that, for any *i *and *j*, either *A*[*i *+ 1, *j *+ 1] = *A*[*i*, *j*] or *A*[*i *+ 1, *j *+ 1] = *A*[*i*, *j*] + 1. That is, the elements along any diagonal of *A *form a non-decreasing sequence of integers. Thus, the computation of *A *can be performed by finding, for all diagonals, the rows in which *A*[*i *+ 1, *j *+ 1] = *A*[*i*, *j*] + 1 ≤ *k*. Such an observation was exploited by Ukkonen [27] in order to obtain a space efficient algorithm for the computation of the edit distance between two strings. Landau and Vishkin [11] cleverly extended the method by Ukkonen to obtain an efficient algorithm that handles the more general problem of string matching with *k *differences. We present their algorithm here, although the asymptotically most efficient one, to date, has been recently obtained by Cole and Hariharan [12].

Let *L*_*d*,*e *_denote the largest row *i *such that *A*[*i*, *j*] = *e *and *j *- *i *= *d*. The definition of *L*_*d*, *e *_implies that *e *is the minimal number of differences between *p*[1, *L*_*d*,*e*_] and the substrings of the text ending at *t*[*L*_*d*,*e *_+ *d*], with *p*[*L*_*d*,*e *_+ 1] ≠ *t*[*L*_*d*,*e *_+ *d *+ 1]. In order to solve the *k *differences problem, we need to compute the values of *L*_*d*,*e *_that satisfy *e *≤ *k*. Assuming that *L*_*d*+1,*e*-1_, *L*_*d*-1,*e*-1 _and *L*_*d*,*e*-1 _have been correctly computed, *L*_*d*,*e *_is computed as follows. Let *row *= *max*(*L*_*d*+1,*e*-1 _+ 1, *L*_*d*-1,*e*-1_, *L*_*d*,*e*-1 _+ 1) and let ℓ be the largest integer such that *p*[*row *+ 1, *row *+ ℓ] = *t*[*d *+ *row *+ 1, *d *+ *row *+ ℓ]. Then, *L*_*d*,*e *_= *row *+ ℓ. The proof of correctness of such a computation is a simple exercise, left to the reader. Moreover, if one makes use of the preprocessing algorithms presented in section 2, *L*_*d*,*e *_can be computed in *O*(1) time as follows:

*L*_*d*,*e *_= *row *+ *Prefix*(*row *+ 1, *row *+ *d *+ 1). Algorithm **SD **gives the needed pseudo-code. We have:

**Theorem 3.1 **[11]*Given a pattern p and a text t, of length m and n, respectively, Algorithm ***SD ***finds all occurrences of p in t with at most k differences in O*(*m *+ *n *+ *nk*) *time, including the preprocessing step*.

### 3.1 The C/C++ library functions

The function below returns all occurrences of a pattern within a text with at most k differences.

Synopsis

#include " k_difference.h"

OCCURRENCES

**k_difference** (char*text, char*pattern, int*k*);

**Arguments**: As in function **k_mismatch**

**Return Values**: As in function **k_mismatch**

1: Algorithm **SD**

2: **Initial Conditions Start Here**

3: **for ***d *:= 0 **to ***n ***do**

4:    *L*[*d*, -1] ← -1

5: **end for**

6: **for ***d *:= -(*k *+ 1) **to **-1 **do**

7:    *L*[*d*, |*d*| - 1] ← |*d*| - 1

8:    *L*[*d*, |*d*| - 2] ← |*d*| - 2

9: **end for**

10: **for ***e *:= -1 **to ***k ***do**

11:    *L*[*n *+ 1, *e*] ← -1

12: **end for**

13: **Initial Conditions End Here**

14: **for ***e *:= 0 **to ***k ***do**

15:    **for ***d *:= -*e ***to ***n ***do**

16:       *row *← max(*L*[*d*, *e *- 1] + 1, *L*[*d *- 1, *e *- 1], *L*[*d *+ 1, *e *- 1] + 1

17:       *row *← min(*row*, *m*)

18:       **if ***row *<*m ***and ***row *+ *d *<*n ***then**

19:          *row *← *row *+ *Prefix*(*row *+ 1, *row *+ *d *+ 1)

20:       **end if**

21:       *L*[*d*, *e*] ← *row*

22:       **if ***L*[*d*, *e*] = *m ***and ***d *+ *m *≤ *n ***then**

23:          **Occurrence Found**

24:       **end if**

25:    **end for**

26: **end for**

### 3.2 The PERL library functions

The function below returns all occurrences of a pattern within a text with at most k differences.

Synopsis

use BSAT::K_Difference;

K_Difference *Text Pattern K*

**Arguments**: As in function **K_Mismatch**

**Return values: **As in function **K_Mismatch**

## 4 Longest common subsequence from fragments

In this section we consider the problem of identifying a longest common subsequence (LCS for short) of two strings *X *and *Y*, using a set *M *of matching fragments. That is, strings of a given length that appear in both *X *and *Y*. We start by reviewing some basic notions about LCS computation and relate them to approximate string matching, discussed in sections 2 and 3. Then, we outline the algorithm presented in [13].

### 4.1 LCS from fragments and edit graphs

**Figure 2:**
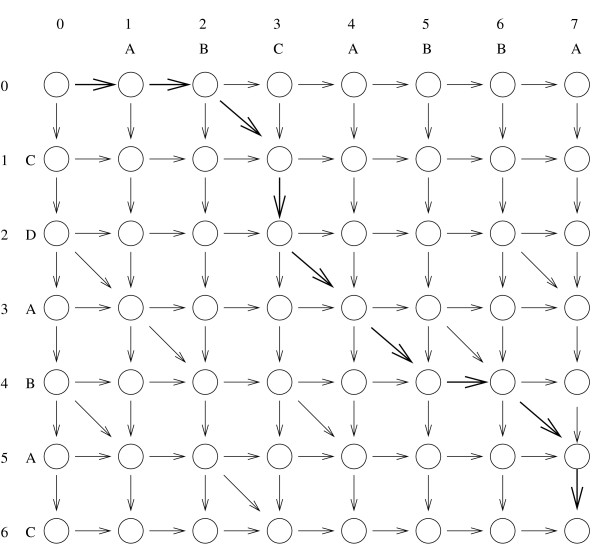
**an edit graph**. An edit graph for the strings *X *= *CDABAC *and *Y *= *ABCABBA*. It naturally corresponds to a **DP **matrix. The bold path from (0, 0) to (6, 7) gives an edit script from which we can recover the LCS between *X *and *Y*.

**Figure 3:**
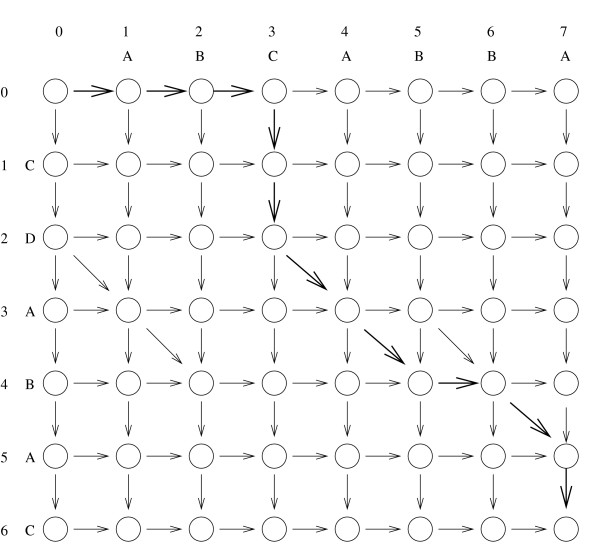
**an edit graph with fragments**. An LCS from Fragments edit graph for the same strings as in Figure 2, where the fragments are the sequences of at least two diagonal edges of Figure 2. The bold path from (0, 0) to (6, 7) corresponds to a minimum-cost path under the Levenshtein edit distance.

It is well known that finding the LCS of *X *and *Y *is equivalent to finding the Levenshtein edit distance between the two strings [4], where the "edit operations" are insertion and deletion of a single character. Those edit operations naturally correspond to the differences of type (b) and (c) introduced in section 3 for approximate string matching. Although there is analogy between approximate string matching and LCS computation, the former can be regarded as a local alignment method as opposed to the latter, that is a global alignment method [1]. Following Myers [28], we phrase the LCS problem as the computation of a shortest path in the edit graph for *X *and *Y*, defined as follows. It is a directed grid graph (see Fig. 2) with vertices (*i*, *j*), where 0 ≤ *i *≤ *n *and 0 ≤ *j *≤ *m*, |*X*| = *n *and |*Y*| = *m*. We refer to the vertices also as *points*. There is a vertical edge from each non-bottom point to its neighbor below. There is a horizontal edge from each non-rightmost point to its right neighbor. Finally, if *X*[*i*] = *Y*[*j*], there is a diagonal edge from (*i *- 1, *j *- 1) to (*i*, *j*). Assume that each non-diagonal edge has weight 1 and the remaining edges weight 0. Then, the Levenshtein edit distance is given by the minimum cost of any path from (0, 0) to (*n*, *m*). We assume the reader to be familiar with the notion of edit script corresponding to the min-cost path and how to recover an LCS from an edit script [28-30]. Our LCS from Fragments problem also corresponds naturally to an edit graph. The vertices and the horizontal and vertical edges are as before, but the diagonal edges correspond to a given set of fragments. Each fragment, formally described as a triple (*i*, *j*, *k*), represents a sequence of diagonal edges from (*i *- *j *- 1) (the *start *point) to (*i *+ *k *- 1, *j *+ *k *- 1) (the *end *point). For a fragment *f*, the start and end points of *f *are denoted by *start*(*f*) and *end*(*f*), respectively. In the example of Figure 3, the fragments are the sequences of at least 2 diagonal edges of Fig. 2. The LCS from Fragments problem is equivalent to finding a minimum-cost path in the edit graph from (0, 0) to (*n*, *m*), where each diagonal edge has weight 0 and each non-diagonal edge has weight 1. The problem has an obvious dynamic programming solution since the graph naturally corresponds to an *nxm *dynamic programming matrix. However, it also falls into the more efficient algorithmic paradigm of Sparse Dynamic Programming [31,32], as discussed in [13] and outlined next.

For a point *p*, define *x*(*p*) and *y*(*p*) to be the *x*- and *y*- coordinates of *p*, respectively. We also refer to *x*(*p*) as the *row *of *p *and *y*(*p*) as the *column *of *p*. Define the diagonal number of *f *to be *d*(*f*) = *y*(*start*(*f*)) - *x*(*start*(*f*)).

We say a fragment *f' *is *left of start*(*f*) if some point of *f' *besides *start*(*f'*) is to the left of *start*(*f*) on a horizontal line through *start*(*f*), or *start*(*f*) lies on *f' *and *x*(*start*(*f'*)) <*x*(*start*(*f*)). (In the latter case, *f *and *f' *are in the same diagonal and overlap.) A fragment *f' *is *above start*(*f*) if some point of *f' *besides *start*(*f'*) is strictly above *start*(*f*) on a vertical line through *start*(*f*).

Define *visl*(*f*) to be the first fragment to the left of *start*(*f*) if such exists, and undefined otherwise. Define *visa*(*f*) to be the first fragment above *start*(*f*) if such exists, and undefined otherwise.

We say that fragment *f *precedes fragment *f' *if *x*(*end*(*f*)) <*x*(*start*(*f'*)) and *y*(*end*(*f*)) <*y*(*start*(*f'*)), i.e. if the end point of *f *is strictly inside the rectangle with opposite corners (0, 0) and *start*(*f'*).

Suppose that fragment *f *precedes fragment *f'*. The shortest path from *end*(*f*) to *start*(*f'*) with no diagonal edges has cost *x*(*start*(*f'*)) - *x*(*end*(*f*)) + *y*(*start*(*f'*)) - *y*(*end*(*f*)), and the minimum cost of any path from (0, 0) to *start*(*f'*) through *f *is that value plus *mincost*_0_(*f*). It will be helpful to separate out the part of this cost that depends on *f *by the definition *Z*(*f*) = *mincost*_0_(*f*) - *x*(*end*(*f*)) - *y*(*end*(*f*)). Note that *Z*(*f*) ≤ 0 since *mincost*_0_(*f*) ≤ *x*(*start*(*f*)) + *y*(*start*(*f*)). The following lemma states that we can compute LCS from fragments by considering only end-points of some fragments rather than all points in the dynamic programming matrix. Moreover, it also gives the appropriate recurrence relations that we need to compute.

**Lemma 4.1 **[13]*For any fragment f and any point p on f, mincost*_0_(*p*) = *mincost*_0_(*start*(*f*)).

*Moreover, mincost*_0_(*f*) *is the minimum of x*(*start*(*f*)) + *y*(*start*(*f*)) *and any of c_p_, c_l_, and c_a _that are defined according to the following:*

*1. If at least one fragment precedes f, c*_*p *_= *x*(*start*(*f*)) + *y*(*start*(*f*)) + min{*Z*(*f'*): *f' precedes f*}.

*2. If visl*(*f*) *is defined, c*_*l *_= *mincost*_0_(*visl*(*f*))+*d*(*f*) - *d*(*visl*(*f*));

*3. If visa*(*f*) *is defined, c*_*a *_= *mincost*_0_(*visa*(*f*)) + *d*(*visa*(*f*)) - *d*(*f*);

### 4.2 Outline of the algorithm

Based on Lemma 4.1, we now present the main steps of the algorithm in [13] computing the required optimal path, given a list *M *of fragments (represented as triples of integers). It uses a sweepline approach where successive rows are processed, and within rows, points are processed from left to right. Lexicographic sorting of (*x*, *y*)-values is needed. The algorithm consists of two main phases, one in which it computes visibility information, i.e., *visl*(*f*) and *visa*(*f*) for each fragment *f*, and the other in which it computes Recurrences (1)–(3) in Lemma 4.1.

Not all the rows and columns need contain a start point or end point, and we generally wish to skip empty rows and columns for efficiency. For any *x *(*y*, resp.), let *C*(*x*) (*R*(*y*), resp.) be the *i *for which *x *is in the *i*-th non-empty column (row, resp.). These values can be calculated in the same time bounds as the lexicographic sorting. From now on, we assume that the algorithm processes only nonempty rows and columns.

For the lexicographic sorting and both phases, we assume the existence of a data structure of type *D *that stores integers *j *in some range [0, *u*] and supports the following operations: (1) insert, (2) delete, (3) member, (4) min, (5) successor: given *j*, the next larger value than *j *in *D*, (6) max: given *j*, find the max value less than *j *in *D*. In our toolkit, *D *is implemented via balanced trees [33]. Therefore, if *d *elements are stored in it, each operation takes *O*(log *d*) time. More complex schemes are proposed and analyzed in [13], yielding better asymptotic performance. With the mentioned data structures, lexicographic sorting of (*x*, *y*)-values can be done in *O*(*d *log *d*) time. In our case *u *≤ *n *+ *m *and *d *≤ |*M*|.

• **Visibility Computation**. We now briefly outline how to compute *visl*(*f*) and *visa*(*f*) for each fragment *f *via a sweepline algorithm. We describe the computation of *visl*(*f*); that for *visa*(*f*) is similar. For *visl*(*f*), the sweepline algorithm sweeps along successive rows. Assume that we have reached row *i*. We keep all fragments crossing row *i *sorted by diagonal number in a data structure *V*. For each fragment *f *such that *x*(*start*(*f*)) = *i*, we record the fragment *f' *to the left of *start*(*f*) in the sorted list of fragments; in this case, *visl*(*f*) = *f'*. Then, for each fragment *f *with *x*(*start*(*f*)) = *i*, we insert *f *into *V*. Finally, we remove fragments f^
 MathType@MTEF@5@5@+=feaafiart1ev1aaatCvAUfKttLearuWrP9MDH5MBPbIqV92AaeXatLxBI9gBaebbnrfifHhDYfgasaacH8akY=wiFfYdH8Gipec8Eeeu0xXdbba9frFj0=OqFfea0dXdd9vqai=hGuQ8kuc9pgc9s8qqaq=dirpe0xb9q8qiLsFr0=vr0=vr0dc8meaabaqaciaacaGaaeqabaqabeGadaaakeaacuWGMbGzgaqcaaaa@2E11@ such that *y*(*end*(f^
 MathType@MTEF@5@5@+=feaafiart1ev1aaatCvAUfKttLearuWrP9MDH5MBPbIqV92AaeXatLxBI9gBaebbnrfifHhDYfgasaacH8akY=wiFfYdH8Gipec8Eeeu0xXdbba9frFj0=OqFfea0dXdd9vqai=hGuQ8kuc9pgc9s8qqaq=dirpe0xb9q8qiLsFr0=vr0=vr0dc8meaabaqaciaacaGaaeqabaqabeGadaaakeaacuWGMbGzgaqcaaaa@2E11@)) = *i*. If the data structure *V *is implemented as a balanced search tree, the total time for this computation is *O*(*M *log *M*).

• **The Main Algorithm**. Again, we use a sweepline approach of processing successive rows. It follows the same paradigm as the Hunt-Szymanski LCS algorithm [34] and the computation of the *RNA *secondary structure (with linear cost functions) [31].

We use another data structure *B *of type *D*, but this time *B *stores column numbers (and a fragment associated with each one). The values stored in *B *will represent the columns at which the minimum value of *Z*(*f*) decreases compared to any columns to the left, i.e. the columns containing an end point of a fragment *f *for which *Z*(*f*) is smaller than *Z*(*f'*) for any *f' *whose end point has already been processed and which is in a column to the left. Notice that, once we fix a row, *D *gives a partition of that row in terms of columns. Within a row, first process any start points in the row from left to right. For each start point of a fragment, compute *mincost*_0 _using Lemma 4.1. Note that when the start point of a fragment *f *is computed, *mincost*_0 _has already been computed for each fragment that precedes *f *and each fragment that is *visa*(*f*) or *visl*(*f*). To find the minimum value of *Z*(*f'*) over all predecessors *f' *of *f*, the data structure *B *is used. The minimum relevant value for *Z*(*f'*) is obtained from *B *by using the max operation to find the max *j *<*y*(*start*(*f*)) in *B*; the fragment *f' *associated with that *j *is one for which *Z*(*f'*) is the minimum (based on endpoints processed so far) over all columns to the left of the column containing *start*(*f*), and in fact this value of *Z*(*f'*) is the

1: Algorithm **FLCS**

2: For each fragment *f*, compute *visl*(*f*) and *visa*(*f*)

3: **for ***i *= *R*(0) to *R*(*n*) **do**

4:    **for **each fragment *f *s.t. *x*(*start*(*f*)) = *i ***do**

5:       *f' *← max on *B *with key *y*(*start*(*f*))

6:       **if ***f' *is defined **then**

7:          compute *cp*

8:       **end if**

9:       **if ***visl*(*f*) is defined **then**

10:          compute *cl*

11:       **end if**

12:       **if ***visa*(*f*) is defined **then**

13:          compute *ca*

14:       **end if**

15:       compute *mincost*(*f*)

16:    **end for**

17:    **for **each fragment *f *s.t. *x*(*start*(*f*)) = *i ***do**

18:       *f' *← max on *B *with key *y*(*end*(*f*)) + 1

19:       **if ***f' *is not defined **or ***Z*(*f*) <*Z*(*f'*) **then**

20:          INSERT *f *into *B *with key *y*(*end*(*f*))

21:       **end if**

22:       **for **each fragment *f' *:= SUCCESSOR(*f*) in *B *such that *Z*(*f'*) ≤ *Z*(*f*) **do**

23:          DELETE(*f'*) from *B*

24:       **end for**

25:    **end for**

26: **end for**

minimum value over all predecessors of *f*.

After any start points for a row have been processed, process the end points. When an end point of a fragment *f *is processed, *B *is updated as necessary if *Z*(*f*) represents a new minimum value at the column *y*(*end*(*f*)); successor and deletion operations may be needed to find and remove any values that have been superseded by the new minimum value. Algorithm **FLCS **gives the pseudo-code of the method just outlined, with the visibility computation omitted for conciseness. In conclusion, we have:

**Theorem 4.2 **[13]*Suppose X *[1 : *n*] *and Y *[1 : *m*] *are strings, and a set M of fragments relating substrings of X and Y is given. One can compute the LCS from Fragments in O*(|*M*|log|*M*|) *time and O*(|*M*|) *space using standard balanced search tree schemes*.

### 4.3 The C/C++ library functions

The function below computes the longest common subsequence from fragments and returns the corresponding alignment.

Synopsis

#include "flcs.h"

ALIGNMENTS

**flcs** (char*X, char*Y, FRAGSET*M*);

**Arguments**:

• *X*: points to a string;

• *Y*: points to a string;

• *M*: point to a FRAGSET_STRUCT, that represents a set of fragments.

**Return Values**: A pointer to ALIGNMENTS_STRUCT, which is defined as:

typedef struct alignments

{

   double *distance*;

   char*X;

   char*Y;

struct alignments*next;

} ALIGNMENTS_STRUCT, *ALIGNMENTS;

where:

• *distance*: is the Levenshtein Distance between strings *X*and *Y*, computed using only fragments;

• *X*: is a pointer to the aligned string *X*, i.e., the string with appropriate spacers inserted;

• *Y*: is a pointer to the aligned string *Y*with appropriate spacers inserted.

One can create a set of fragments from all the matching *k*-tuples between *X*and *Y*, using the function:

FRAGSET

**fragset_create_ktuples **(char*X, char*Y, int*k*);

where:

• *X*: points to string;

• *Y*: points to a string;

• *k*: is the fragment length.

Auxiliary functions destroying, creating or incrementally updating a set of fragments are the following:

void

**fragset_destroy**(FRAGSET* fragset*);

FRAGSET

**fragset_create**(int*max_cardinality);

int

**fragset_frag_add**(FRAGSET *fragset*, int *i*, int* j*, int* length*);

where

• *fragset*:points to FRAGSET_STRUCT;

• *i*: fragment starting position in the first string *X*;

• *j*: fragment starting position in the second string *Y*;

• *length*: fragment length.

### 4.4 The PERL library functions

The function FLCS computes the longest common subsequence from fragments. It returns the corresponding alignment.

Synopsis

use BSAT::FLCS;

FLCS *X Y Frags*

**Arguments**:

• *X*: is a scalar containing string X.

• *Y*: is a scalar containing string Y.

• *Frags*: is a hash reference (see below).

**Return values: **FLCS returns a hash corresponding to the alignment between X and Y:

my %alignment = (

   distance => 0,

   X => "",

   Y => "");

where:

• distance: is a scalar containing the Levenshtein Distance between strings *X*and *Y*, computed using only fragments;

• X: is a scalar containing the alignment string X;

• Y: is a scalar containing the alignment string Y.

The hash reference Frags is defined as:

my %Frags = (

   K => 0,

   Set => ());

where:

• K: is a scalar giving the fragment length;

• Set: is an array of three elements (*i*, *j*, *length*) specifying a fragment.

## 5 Edit distance with gaps

### 5.1 The dynamic programming recurrences

We refer to the edit operations of substitution of one symbol for another (point mutation), deletion of a single symbol, and insertion of a single symbol as *basic operations*. They are related in a natural way to the differences introduced in section 3. Let a *gap *be a consecutive set of deleted symbols in one string or inserted symbols in the other string. With the basic set of operations, the cost of a gap is the sum of the costs of the individual insertions or deletions which compose it. Therefore, a gap is considered as a sequence of homogeneous elementary events (insertion or deletion) rather than as an elementary event itself. But, both theoretic and experimental considerations [1,14,35], suggest that the cost *w*(*i*, *j*) of a generic gap *X*[*i*, *j*] must be of the form

*w*(*i*, *j*) = *f*^1^(*X*[*i*]) + *f*^2^(*X*[*j*]) + *g*(*j *- *i*)

where *f*^1 ^and *f*^2 ^are the costs of breaking the string at the endpoints of the gap and *g *is a function that increases with the gap length.

In molecular biology, the most likely choices for *g *are affine or concave functions of the gap lengths, e.g., *g*(ℓ) = *c*_1 _+ *c*_2_ℓ or *g*(ℓ) = *c*_1 _+ *c*_2 _log ℓ, where *c*_1 _and *c*_2 _are constants. With such a choice of *g*, the cost of a long gap is less than or equal to the sums of the costs of any partition of the gap into smaller gaps. That is, each gap is treated as a unit. Such constraint on *g *induces a constraint on the function *w*. Indeed, *w *must satisfy the following inequality, known as *concave Monge condition *[7]:

*w*(*a*, *c*) + *w*(*b*, *d*) ≥ *w*(*b*, *c*) + *w*(*a*, *d*) for all *a *<*b *and *c *<*d*.

an extremely useful inequality that yields speed-ups in Dynamic Programming [7].

The gap sequence alignment problem can be solved by computing the following dynamic programming equation (*w' *is a cost function analogous to *w*):

*D*[*i*, *j*] = min{*D*[*i *- 1, *j *- 1] + *sub*(*X*[*i*], *Y*[*j*]), *E*[*i*, *j*], *F*[*i*, *j*]}

where

E[i,j]=min⁡0≤k≤j−1{D[i,k]+w(k,j)},
 MathType@MTEF@5@5@+=feaafiart1ev1aaatCvAUfKttLearuWrP9MDH5MBPbIqV92AaeXatLxBI9gBaebbnrfifHhDYfgasaacH8akY=wiFfYdH8Gipec8Eeeu0xXdbba9frFj0=OqFfea0dXdd9vqai=hGuQ8kuc9pgc9s8qqaq=dirpe0xb9q8qiLsFr0=vr0=vr0dc8meaabaqaciaacaGaaeqabaqabeGadaaakeaacqWGfbqrdaWadaqaaiabdMgaPjabcYcaSiabdQgaQbGaay5waiaaw2faaiabg2da9maaxababaGagiyBa0MaeiyAaKMaeiOBa4galeaacqaIWaamcqGHKjYOcqWGRbWAcqGHKjYOcqWGQbGAcqGHsislcqaIXaqmaeqaaOWaaiWabeaacqWGebardaWadaqaaiabdMgaPjabcYcaSiabdUgaRbGaay5waiaaw2faaiabgUcaRiabdEha3naabmaabaGaem4AaSMaeiilaWIaemOAaOgacaGLOaGaayzkaaaacaGL7bGaayzFaaGaeiilaWcaaa@52D2@

F[i,j]=min⁡0≤l≤i−1{D[i,j]+w′(l,i)},
 MathType@MTEF@5@5@+=feaafiart1ev1aaatCvAUfKttLearuWrP9MDH5MBPbIqV92AaeXatLxBI9gBaebbnrfifHhDYfgasaacH8akY=wiFfYdH8Gipec8Eeeu0xXdbba9frFj0=OqFfea0dXdd9vqai=hGuQ8kuc9pgc9s8qqaq=dirpe0xb9q8qiLsFr0=vr0=vr0dc8meaabaqaciaacaGaaeqabaqabeGadaaakeaacqWGgbGrdaWadaqaaiabdMgaPjabcYcaSiabdQgaQbGaay5waiaaw2faaiabg2da9maaxababaGagiyBa0MaeiyAaKMaeiOBa4galeaacqaIWaamcqGHKjYOcqWGSbaBcqGHKjYOcqWGPbqAcqGHsislcqaIXaqmaeqaaOWaaiWabeaacqWGebardaWadaqaaiabdMgaPjabcYcaSiabdQgaQbGaay5waiaaw2faaiabgUcaRiqbdEha3zaafaWaaeWaaeaacqWGSbaBcqGGSaalcqWGPbqAaiaawIcacaGLPaaaaiaawUhacaGL9baacqGGSaalaaa@52DE@

*sub *is a symbol substitution cost matrix and the initial conditions of recurrence (6) are *D*[*i*, 0] = *w'*(0, *i*), 1 ≤ *i *≤ *m *and *D*[0, *j*] = *w*(0, *j*), 1 ≤ *j *≤ *n*.

We observe that the computation of recurrence (6) consists of *n *+ *m *interleaved subproblems that have the following general form: Compute

E[j]=min⁡0≤k≤j−1{D[k]+w(k,j)},j=1,⋯,n,
 MathType@MTEF@5@5@+=feaafiart1ev1aaatCvAUfKttLearuWrP9MDH5MBPbIqV92AaeXatLxBI9gBaebbnrfifHhDYfgasaacH8akY=wiFfYdH8Gipec8Eeeu0xXdbba9frFj0=OqFfea0dXdd9vqai=hGuQ8kuc9pgc9s8qqaq=dirpe0xb9q8qiLsFr0=vr0=vr0dc8meaabaqaciaacaGaaeqabaqabeGadaaakeaafaqabeqacaaabaGaemyrau0aamWaaeaacqWGQbGAaiaawUfacaGLDbaacqGH9aqpdaWfqaqaaiGbc2gaTjabcMgaPjabc6gaUbWcbaGaeGimaaJaeyizImQaem4AaSMaeyizImQaemOAaOMaeyOeI0IaeGymaedabeaakmaacmqabaGaemiraq0aamWaaeaacqWGRbWAaiaawUfacaGLDbaacqGHRaWkcqWG3bWDdaqadaqaaiabdUgaRjabcYcaSiabdQgaQbGaayjkaiaawMcaaaGaay5Eaiaaw2haaiabcYcaSaqaaiabdQgaQjabg2da9iabigdaXiabcYcaSiabl+UimjabcYcaSiabd6gaUbaacqGGSaalaaa@57AF@

*D*[0] is given and for every *k *= 1,..., *n*, *D *[*k*] is easily computed from *E*[*k*]. We now concentrate on a general algorithm computing (9).

### 5.2 The GG algorithm

From now on, unless otherwise specified, we assume that *w *satisfies the concave Monge condition (5). An important notion related to concave Monge condition is concave total monotonicity of an *s *× *p *matrix *A*. *A *is *concave totally monotone *if and only if

*A*[*a*, *c*] ≤ *A*[*b*, *c*] ⇒ *A*[*a*, *d*] ≤ *A*[*b*, *d*].

for all *a *<*b *and *c *<*d*.

It is easy to check that if *w *is seen as a two-dimensional matrix, the concave Monge condition implies concave total monotonicity of *w*. Notice that the converse is not true. Total monotonicity and Monge condition of a matrix *A *are relevant to the design of algorithms because of the following observations. Let *r*_*j *_denote the row index such that *A*[*r*_*j*_, *j*] is the minimum value in column *j*. Concave total monotonicity implies that the minimum row indices are nonincreasing, i.e., *r*_1 _≥ *r*_2 _≥ ... ≥ *r*_*m*_. We say that an element *A*[*i*, *j*] is *dead *if *i *≠ = *r*_*j *_(i.e., *A*[*i*, *j*] is not the minimum of column *j*). A submatrix of *A *is dead if all of its elements are dead.

Let *B*[*i*, *j*] = *D*[*i*] + *w*(*i*, *j*), for 0 ≤ *i *≤ *j *≤ *n*. We say that *B*[*i*, *j*] is *available *if *D*[*i*] is known and therefore *B*[*i*, *j*] can be computed in constant time. That is, *B*[*i*, *j*] is available only when the column minima for columns 1, 2,..., *i *have been found. We say that *B *is *on-line*, since its entries become available as the computation proceeds.

The computation of recurrence (9) reduces to the identification of the column minima in an on-line upper triangular matrix *B*. One can easily show that when *w *satisfies the concave Monge condition, *B *is totally monotone. We make use of this fact to obtain an efficient algorithm.

The algorithm outlined here finds column minima one at a time and processes available entries so that it keeps only possible candidates for future column minima. In the concave case, we use a stack to maintain the candidates. The algorithm can be sketched as follows (proof of correctness can be found in [15])

For each *j*, 2 ≤ *j *≤ *n*, we find the minimum at column *j *as follows. Assume that (*i*_1_, *h*_1_),..., (*i*_*k*_, *h*_*k*_) are on the stack ((*i*_1_, *h*_1_) is at the top of the stack). Initially, (0, *n*) is on the stack. The invariant on the stack elements is that in submatrix *B*[0 : *j *- 2, *j *: *n*] row *i*_*r*_, for 1 ≤ *r *≤ *k*, is the best (gives the minimum) in the column interval [*h*_*r*-1 _+ 1, *h*_*r*_] (assumingh *h*_0 _+ 1 = *j*). By the concave total monotonicity of *B*, *i*_1_,..., *i*_*k *_are nonincreasing. Thus the minimum at column *j *is the minimum of *B*[*i*_1_, *j*] and *B*[*j *- 1, *j*].

Now we update the stack with row *j *- 1 as follows.

(**GG1) **If *B*[*i*_1_, *j*] ≤ *B*[*j *- 1, *j*], row *j *- 1 is dead by concave total monotonicity. If *h*_1 _= *j*, we pop the top element because it will not be useful.

(**GG2) **If *B*[*i*_1_, *j*] > *B*[*j *- 1, *j*], we compare row *j *- 1 with row *i*_*r *_at *h*_*r *_(i.e., *B*[*i*_*r*_, *h*_*r*_] vs. *B*[*j *- 1, *h*_*r*_]), for *r *= 1, 2,..., until row *i*_*r *_is better than row *j *- 1 at *h*_*r*_. If row *j *- 1 is better than row *i*_*r *_at *h*_*r*_, row *i*_*r *_cannot give the minimum for any column because row *j *- 1 is better than row *i*_*r *_for column *l *≤ *h*_*r *_and row *i*_*r*+1 _is better than row *i*_*r *_for column *l *> *h*_*r*_. We pop the element (*i*_*r*_, *h*_*r*_) from the stack and continue to compare row *j *- 1 with row *i*_*r*+1_. If row *i*_*r *_is better than row *j *- 1 at *h*_*r*_, we need to find the border of the two rows *j *- 1 and *i*_*r*_, which is the largest *h *<*h*_*r *_such that row *j *- 1 is better than row *i*_*r *_for column *l *≤ *h*; i.e., finding the zero *z *of *f*(*x*) = *B*[*j *- 1, *x*] - *B*[*i*_*r*_, *x*] = *w*(*j *- 1, *x*) - *w*(*i*_*r*_, *x*) + (*D*[*j *- 1] - *D*[*i*_*r*_]), then *h *= ⌊*z*⌋. If *h *≥ *j *+1, we push (*j *- 1, *h*) into the stack.

In the pseudo-code of Algorithm **GG**, let *I*(*top*) and *H*(*top*) denote (*i*_1_, *h*_1_). Moreover, let *CLOSEST*(*j *- 1, *I*(*top*)) be a function that returns the zero of *f*(*x*) (defined in step **GG2**) closest to *j *- 1. Notice that, using the monotonicity conditions on *w*, *CLOSEST*(*j *- 1, *I*(*top*)) can be computed in *O*(log *n*) time. Moreover, we say that *f *satisfies the *closest zero property *if such a zero can be computed in constant time. We also notice that when *w *is a linear function, *f *obviously satisfies the closet zero property. Moreover, for linear functions, lines 9–20 of Algorithm **GG **become useless since only one element can be on the stack: the winner (the minimum) of the comparison on line 5 of the algorithm. We have:

**Theorem 5.1 ***Recurrence (9) can be computed in O*(*n *log *n*) *time when w satisfies the concave Monge conditions. The time reduces to O*(*n*) *when the closet zero property is satisfied or w is linear. Therefore, given two strings X and Y, their edit distance with gaps can be computed in time O*(*nm *log max(*n*, *m*)) *time, when both w and w' satisfy the concave Monge conditions and O*(*nm*) *time when both functions satisfy the closest zero property or are affine gap costs*.

Two remarks are in order regarding the implementation of the **GG **algorithm provided here:

1: Algorithm **GG**

2: **push **(0, *n*) on *S*

3: **for ***j *:= 2 **to ***n ***do**

4:    ℓ ← *I*(*top*)

5:    **if ***B*[*j *- 1, *j*] ≥ *B*[ℓ, *j*] **then**

6:       min is *B*[ℓ, *j*]

7:    **else**

8:       min is *B*[*j *- 1, *j*]

9:       **while ***S *≠ ∅ **and ***B*[*j *- 1, *j*] ≤ *B*[*I*(*top*); *H*(*top*)] **do**

10:          **pop**

11:       **end while**

12:       **if ***S *= ∅ **then**

13:          **push **(*j *- 1, *n*)

14:       **else**

15:          *h *← *CLOSEST*(*j *- 1, *I*(*top*))

16:          **push **(*j *- 1, *h*)

17:       **end if**

18:    **end if**

19:    **if ***H*(*top*) = *j ***then**

20:       **pop**

21:    **end if**

22: **end for**

(a) It takes in input a character substitution matrix. Such a matrix could be one of the well known PAM [36] or BLOSUM [37,38] matrices. However, those matrices have been designed for maximization problems, while we have stated our alignment problem as a minimization problem. Therefore, in order to use those matrices, we need to change the sign of each entry, i.e., take its dual.

(b) It takes in input two default gap cost functions, one affine and the other concave: *g*(ℓ) = *c*_1 _+ *c*_2_ℓ and *g*(ℓ) = *c*_1 _+ *c*_2 _log ℓ, where *c*_1 _and *c*_2 _are constants. In this case, the closet zero property holds and the program uses this condition to avoid the binary search. However, the user can also specify a concave cost function by simply providing a pointer to the excutable computing it. In this case, the binary search is used.

### 5.3 The C/C++ library functions

The function below computes the edit distance between two strings, using convex or affine gap costs. It returns the corresponding alignment.

Synopsis

#include "edit_distance_gaps.h"

ALIGNMENTS

**edit_distance_gaps**(char*X, char*Y, WEIGHT* Xw*, WEIGHT *Yw*,, MATRIX* substitution*);

**Arguments**:

• *X*: points to a string;

• *Y*: points to a string;

• *Xw*: is a pointer to a WEIGHT_STRUCT;

• *Yw*: is a pointer to a WEIGHT_STRUCT;

• *substitution*: is a pointer to MATRIX_STRUCT, a data structure (detailed below) defining an upper triangular substitution cost matrix.

WEIGHT_STRUCT defines a generic cost function for gaps, as follows:

typedef struct weight

{

   int* type*;

double* Wa*, *Wg*, *base*;

double (*w)(int *l*, int* k*);

} WEIGHT_STRUCT, *WEIGHT;

The *type* is a mendatory field that takes two values:F_AFFINE and F_CONCAVE. In both cases, the total of gap opening and closing costs, i.e., *Wg*, and the gap extension cost, i.e., *Wa*, need also be specified. Then, the affine function is *W*_*a *_+ *W*_*g*_ℓ, for a gap of length ℓ. For the concave cost function, we can use the default *W*_*a *_+ *W*_*g*_*log*_*base*_(ℓ), where the base of the logarithm must also be specified. One can also use a user-defined concave cost function *w *by specifying a pointer to a function defined as:

double

**weight_function**(int *l*, int* k*);

MATRIX_STRUCT defines a generic cost substitution matrix, as follows:

typedef struct matrix

{

   char*alphabet;

   int* size*

   double**matrix

} MATRIX_STRUCT, *MATRIX;

where *alphabet* is a pointer to the alphabet array (case insensitive) of cardinality *size*. The last field *matrix*is a pointer an upper triangular symbol substitution cost matrix. In case one wants to use the default matrix, i.e., match values 0 and mismatch 1, it suffices to set filed *size *equal to zero.

**Return Values**: A pointer to ALIGNMENTS_STRUCT, which is defined as in section 4.3, except that *distance* now refers to the edit distance with gaps.

### 5.4 The Perl library functions

The **Edit_Distance_Gap **computes the edit distance with gaps between two strings.

Synopsis

use BSAT::Edit_Distance_Gaps;

Edit_Distance_Gaps *X Y Xw Yw Substitution*

**Arguments**:

• *X*: is a scalar containing string X;

• *Y*: is a scalar containing string Y;

• *Xw*: is a hash reference defined below;

• *Yw*: is a hash reference defined below;

• *Yw*: is a list reference containing the

• *Substitution*: is a list reference containing an upper triangular symbol substitution cost matrix. If undefined, the default values are used, as in section 5.3;

• *Alphabet*: is a list reference containing the characters of alphabet (case insensitive). If undefined, the default values are used, as in section 5.3.

Xw is defined as (Yw is analogous):

my %Xw = (

   *Type *=> "",

   *Wa *=> 0,

   *Wg *=> 0,

   *Base *=> 0,

   *w *=> \&*custom_fuction*);

where the fields are as in the specification of the cost function in section 5.3.

**Return values**: **Edit_Distance_Gaps **returns an hash corresponding to the computed alignment and it is defined as in section 4.4, except the distance is now the value of the edit distance with gaps:

my %alignment = (

   distance => 0,

   X => "",

   Y => "");

## 6 Filtering, statistical scores and model organism generation

In this section we outline the filtering and statistical functions present in the system, starting with the filter. Let *O*_1_,...,*O*_*s *_be the output of algorithm **SM **on the pattern strings *p*_1_,...,*p*_s _and text strings *t*_1_,...,*t*_*s*_, respectively. We assume that the algorithm has been used with the same value of *k *in all *s *instances. The procedure takes in input the sets *O*_*i *_and *t*_*i*_, 1 ≤ *i *≤ *s*, and a threshold parameter *th*. It returns a set *W *consisting of all strings in *O*_*i *_that appear in at least *th *of the text strings. Since each *O*_*i *_consists of the occurrences of a pattern *p*_*i *_in *t*_*i*_, with mismatches, *W *corresponds to a set of strings representing common occurrences of all patterns in the text strings, i.e., it is a consensus set. The algorithmic details yielding an efficient implementation of the filtering operation are straightforward and therefore omitted.

We now turn to the z-score. The assessment of the statistical significance of the occurrences of a set of strings *W *in a set of text strings *t*_1_,...,*t*_*s *_is a well established procedure for analysis of biological sequences, in particular via z-score functions [18]. Intuitively, the value of the z-score for a set of strings *W *gives an indication of how relevant are the occurrences of the strings in *W *in the text strings *t*_1_,...,*t*_*s*_, with respect to "a random event" as characterized by a background model. We limit ourselves to give formal definitions and for the case in which *W *contains only one string and *s *= 1. For the generalization to the case in which *W *contains more than one string and the rather involved algorithmic details, the reader is referred to [19].

Let *p *be a string and let *X *be a set of random strings, generated according to some " background probabilistic model", usually a Markov Source. Let *X*_*p *_be the random variable indicating the number of occurrences of *p *in *X *and let *E*(*X*_*p*_) and *σ*(*X*_*p*_) be the mean and standard deviation, respectively. Then, the *z-score *associated with *p *is

zp=Np−E(Xp)σ(Xp)
 MathType@MTEF@5@5@+=feaafiart1ev1aaatCvAUfKttLearuWrP9MDH5MBPbIqV92AaeXatLxBI9gBaebbnrfifHhDYfgasaacH8akY=wiFfYdH8Gipec8Eeeu0xXdbba9frFj0=OqFfea0dXdd9vqai=hGuQ8kuc9pgc9s8qqaq=dirpe0xb9q8qiLsFr0=vr0=vr0dc8meaabaqaciaacaGaaeqabaqabeGadaaakeaacqWG6bGEdaWgaaWcbaGaemiCaahabeaakiabg2da9maalaaabaGaemOta40aaSbaaSqaaiabdchaWbqabaGccqGHsislcqWGfbqrdaqadaqaaiabdIfaynaaBaaaleaacqWGWbaCaeqaaaGccaGLOaGaayzkaaaabaacciGae83Wdm3aaeWaaeaacqWGybawdaWgaaWcbaGaemiCaahabeaaaOGaayjkaiaawMcaaaaaaaa@402E@

where *N*_*p *_is the number of occurrences of *p *in the strings in *X*. Notice that *z*_*p *_gives the number of standard deviations by which the observed value *N*_*p *_exceeds its expected value. It is normalized so that it has mean zero and standard deviation one, so that it can be used to compare the z-score of different strings.

The module that computes the z-score in our system takes in input the set *W *output by the filtering function, the text strings *t*_1_,...,*t*_*s *_and a model, i.e., a table encoding a Markov source of order 3, together with additional information needed for the computation of the variance (see Appendix A in [39]). The software computing the z-score is a specialization of the software of Sinha and Tompa for the computation of the z-score in YMF, that is designed to work for motifs (a concise and general encoding of a set of strings). As in their case, the code is designed to work only for DNA sequences. Therefore, care must be taken in computing the number of occurrences of a string *p *in a string *t*. In fact, one must count occurrences on both DNA strands. That is done by including, for each string in the input set *W*, its reverse complement.

Two model organisms are available, Human and Yeast, as they are given by the YMF software distribution of Sinha and Tompa [39]. Moreover, via the function that generates a model organism, the user can specify a new model for her/his sequences. Details on input formats for the model are given in the User Guide.

### 6.1 The C/C++ library functions

The function below computes the z-score value of a set of patterns (all of the same length) with respect to a set of sequences (all of the same length). It works for DNA only.

Synopsis

#include "z_score.h"

double

**z score **(char**patterns, char**texts, char*organismpath);

**Arguments**:

• *patterns*: is a column vector, each item points to a pattern string. The last item point to NULL;

• *texts*: is a column vector, each item points to a text string. The last item point to NULL;

• *organismpath*: it is the path to the file containing all probabilistic information for an organism.

Return Values

Upon successful completion **z_score**return a double value, corresponding to z-score.

The function below generates a Markov model of order 3, from a set of strings. It works for DNA only.

Synopsis

#include " model_generatation.h"

int

**model_generatation **(char**strings, char*path, char*organism);

**Arguments**:

• *strings*: is a column vector, each item points to a string. The last item point to NULL;

• path: is a output path;

• organism: is the organism name;

Return Values

**model_generation** returns zero if the computation is completed successfully and 1 otherwise.

### 6.2 The Perl library functions

The function below performs a filtering operation on a set of sequences.

use BATS::Filter;

Filter *files hits score Hitsthreshold Filesthreshold*

**Arguments**:

• *files*: is an array of strings containing the filenames.

• *hits*: is a hash reference containing number of hits for each occurrence per file.

• *score*: is a hash reference containing number of errors for each occurrence.

• *Filesthreshold*: is a scalar containing the minimum number of hits on which occurrences need to be present.

• *Filesthreshold*: is a scalar containing the minimum percentage of files on which occurrences need to be present.

**Return values **Filter returns an array containing indices of hits that satisfy the threshold.

The function below computes the z-score value of a set of patterns (all of the same length) with respect to a set of sequences (all of the same length). It works for DNA only.

Synopsis

use BATS::Z_Score;

Z_Score *patters texts organismpath*

**Arguments**:

• *patterns*: is an array of strings containing the set of patterns;

• *sequences*: is an array of strings containing the text strings;

• *organismpath*: it is the path to the file containing all probabilistic information for an organism.

Return values:

Z_Score returns a scalar containing the z-score value of the pattern set.

The function below generates a Markov model of order 3, from a set of strings. It works for DNA only.

Synopsis

use BATS::Model_Generation;

Model_Generatation *strings path organism*

**Arguments**:

• *strings*: is an array of strings;

• *path*: is a scalar containing the string of the output path;

• *organism*: points to the string containing the name of the organism;

**Return values: **Model_Generation returns a scalar containing 0 if the computation is completed successfully and 1 otherwise.

## 7 Conclusion

We have presented a software library for some basic global and local sequence alignment tasks. Moreover, procedures to assess the statistical significance of the occurrence of a set of DNA pattern strings in a set of DNA text strings has also been provided. Although none of the presented algorithms is new, this the first software library that provides their implementation in one consistent and ready to use package.
